# Determinants of carcass traits and pricing in crossbred beef cattle under a cooperative production system in tropical Thailand

**DOI:** 10.5713/ab.250993

**Published:** 2026-06-01

**Authors:** Suphawida Hiranrueang, Thanathip Suwanasopee, Mauricio A. Elzo, Skorn Koonawootrittriron

**Affiliations:** 1 Department of Animal Science, Faculty of Agriculture, Kasetsart University, Bangkok, Thailand; 2 Tropical Animal Genetic Special Research Unit (TAGU), Kasetsart University, Bangkok, Thailand; 3 Department of Animal Sciences, University of Florida, Gainesville, FL, USA

**Keywords:** Breed Group, Carcass Quality, Cooperative Production System, Marbling Score, Pricing, Sex

## Abstract

**Objective:**

Quantitative evidence linking carcass traits to pricing under cooperative beef production systems in tropical regions is still scarce. This study aimed to identify the biological and management determinants of carcass traits and their economic implications in crossbred beef cattle raised under a cooperative system in tropical Thailand.

**Methods:**

The carcass and pricing records from 5,531 crossbred finished cattle (3,109 steers and 2,422 females) across six breed groups and 217 farms were examined. Fixed effects in general linear models were farm–year–season (FYS), breed group, sex, and physiological maturity (number of permanent incisors; teeth). Marbling score (MS) and cover fat thickness (COVF) were additionally fitted for pricing traits.

**Results:**

Carcass and pricing traits were strongly influenced by FYS, breed group, sex, Teeth, MS, and COVF (p<0.001). Each additional pair of teeth increased live weight (+15.28 kg) and cold carcass weight (+7.93 kg) but reduced price per carcass weight (−0.53 THB/kg). MS was the strongest economic driver; a one-unit increase increased carcass price by 12.34 THB/kg and live value by 5,889 THB/head. COVF had positive but smaller effects. Charolais crossbreds exhibited the highest carcass mass, Wagyu had crossbreds the greatest marbling, and Brahman crossbreds had the highest dressing percentage.

**Conclusion:**

Carcass value is driven by two pathways: carcass mass determines total return, and marbling drives price premiums, providing a biologically grounded framework for optimizing breed selection, management, and slaughter strategies in tropical cooperative systems.

## INTRODUCTION

Global demand for high-quality beef has increased substantially, driven by consumer emphasis on eating quality and perceived value, with premium prices for products that consistently meet these expectations [1,2]. Carcass traits, including live weight (LW), carcass weight, dressing percentage, marbling score (MS), and cover fat thickness (COVF), are central to market valuation because they directly influence tenderness, juiciness, flavor, and saleable yield [2,3]. Among these, intramuscular fat deposition, reflected by marbling, is a primary determinant of eating quality and the basis of grading systems in major beef-producing countries [4]. Importantly, marbling is regulated by biological processes that are partly independent of overall growth, reinforcing its distinct role in value differentiation [3,5].

Genetic background is a major source of variation in carcass characteristics. In tropical production systems, crossbreeding between *Bos indicus* (e.g., Brahman and Thai native cattle) and *Bos taurus* breeds (e.g., Angus, Charolais, and Wagyu) is widely used to balance adaptability, growth performance, and carcass quality [6]. Charolais-derived cattle typically exhibit superior growth and carcass yield, whereas Wagyu and Angus are associated with enhanced marbling and meat quality [7,8]. These differences reflect variation in muscle accretion and adipogenesis, which regulate carcass composition and market value, and may also be influenced by nutritional strategies affecting growth performance and carcass quality [5,9,10]. In addition, sex and physiological maturity influence carcass traits through their effects on growth dynamics and nutrient partitioning [11–13], while COVF contributes to carcass preservation and grading outcomes, although excessive fat may reduce processing efficiency [14].

In Thailand, beef demand is characterized by a dual-market structure comprising a traditional segment that prioritizes affordability and lean meat, and a rapidly expanding premium segment driven by eating quality attributes, particularly marbling. These contrasting consumer preferences directly influence carcass grading systems and price differentiation within cooperative production systems. In this context, cooperative pricing aims to ensure equity by establishing a transparent, consistent carcass-based pricing system in which payments are determined by objectively measured carcass traits, thereby aligning economic returns with biological performance among producers. Given that fattened cattle represent only a small proportion of the national herd [15], such cooperative systems play a critical role in standardizing production practices, improving market access, and facilitating the implementation of carcass-based pricing mechanisms under smallholder production conditions. Recent developments in carcass grading technologies and standardized evaluation systems further emphasize the importance of objective and transparent pricing frameworks in modern beef markets.

Despite these developments, quantitative evidence linking carcass traits with pricing outcomes in cooperative systems remains limited. Previous studies have generally examined carcass traits or pricing separately, with limited integration of biological, environmental, and economic determinants under commercial production conditions. This gap restricts understanding of how growth-related traits (e.g., carcass mass) and quality-related traits (e.g., marbling) jointly determine price formation, particularly in tropical systems.

To address this limitation, this study adopted an integrated biological–economic perspective in which carcass value is determined through two complementary pathways: (1) a mass-driven pathway associated with growth and carcass weight, and (2) a quality-driven pathway associated with intramuscular fat deposition. Therefore, the objectives were to: (1) evaluate the effects of farm–year–season (FYS), breed group (BG), sex, physiological maturity (Teeth), MS, and COVF on carcass traits and pricing; and (2) quantify the relationships between carcass traits and economic outcomes. This approach provides a mechanistic understanding of how biological and management factors jointly influence carcass composition and price formation, supporting data-driven decision-making in tropical cooperative beef production systems.

## MATERIALS AND METHODS

### Animals and traits recorded

Data were collected from 11,189 fattened cattle (7,284 castrated males [steers] and 3,898 females; no intact males) raised on 217 farms affiliated with the Beef Cluster Cooperative Limited (Max Beef), Nakhon Pathom province, Thailand (14.05373°N, 99.98996°E), between 2019 and 2023. All farms operated under a standardized cooperative production system.

Recorded variables included animal identification, farm identification, BG, sex (steers vs. females), number of permanent incisor pairs (Teeth), and LW.

Following data screening, 5,531 cattle (3,109 steers and 2,422 females; no intact males) with complete and reliable records were retained for analysis, while records with missing or inconsistent information were excluded. Physiological maturity (age class) was approximated using the number of permanent incisor pairs (Teeth; 0 to 4 pairs) following Pace and Wakeman [16].

BGs were classified as Angus crossbred (ANGx; n = 484), Beefmaster crossbred (BFMx; n = 13), Brahman crossbred (BRAx; n = 46), Charolais crossbred (CHAx; n = 1,586), Holstein crossbred (HOLx; n = 1,801), and Wagyu crossbred (WAGx; n = 1,601). All male animals were castrated and managed as steers under standardized cooperative practices; therefore, sex effects in the analysis represent differences between steers and females.

All cattle were intensively finished for a minimum of 8 months prior to slaughter. Slaughter eligibility required a minimum LW of 550 kg. Female cattle were non-pregnant and non-lactating at the time of slaughter.

### Slaughter procedures and carcass measurements

Finished cattle were transported to a good manufacturing practice (GMP)-certified commercial slaughterhouse in Ratchaburi province and handled to minimize stress. Animals were fasted for 10 to12 h with ad libitum access to water before slaughter. Standard commercial procedures were followed for stunning, bleeding, dressing, and carcass splitting.

Hot carcass weight (HCW) was recorded prior to chilling. Carcasses were chilled at 4°C for 7 days, after which cold carcass weight (CCW) was measured, reflecting carcass yield and quality [17]. The left carcass side was ribbed between the 12th and 13th ribs. MS was assessed on the *longissimus dorsi* according to the ACT 6001–2004 Beef Standard [18]. COVF was measured at three-fourths of the distance across the ribeye from the chine bone using a millimeter-graduated ruler by a trained grader.

### Pricing criteria

Cooperative pricing was primarily based on BG and MS to determine the price per kilogram of cold carcass (PCKG), consistent with market demand and grading standards. For dairy-influenced cattle, Teeth were also considered. Live cattle price per head (PLCAT) was calculated as:


(1) PLCAT=PCKG×CCW

Price per live-weight kilogram (PLKG) was then computed as:


(2) $$\text{PLKG}=\frac{\text{PLCAT}}{\text{LW}}$$

The final dataset included farm ID, slaughter date, BG, sex, Teeth, LW, HCW, CCW, hot carcass percentage (HCP), cold carcass percentage (CCP), MS, COVF, PCKG, PLCAT, and PLKG.

### Statistical analysis

#### Descriptive statistics and contemporary grouping

Descriptive statistics, including minimum, maximum, mean, standard deviation, and coefficient of variation, were calculated for all traits. Animals were assigned to FYS contemporary groups based on slaughter date, with seasons defined as winter (November to February), summer (March to June), and rainy (July to October). In total, 909 FYS contemporary groups were constructed to account for combined management, environmental, and temporal variation.

#### Model for carcass quality traits

Carcass traits, including LW, HCW, CCW, HCP, CCP, MS, and COVF, were analyzed using the general linear model (GLM) procedure in SAS (SAS OnDemand for Academics; SAS Institute) [19]:


(3) $${\text{y}}_{\text{ijkl}}=\mu +{\text{FYS}}_{\text{i}}+{\text{BG}}_{\text{j}}+{\text{Sex}}_{\text{k}}+{\text{b}}_{\text{l}}(\text{Teeth})+{\text{e}}_{\text{ijkl}}$$

Pricing traits, including PCKG, PLCAT, and PLKG, were analyzed using:


(4) $${\text{y}}_{\text{ijkl}}=\mu +{\text{FYS}}_{\text{i}}+{\text{BG}}_{\text{j}}+{\text{Sex}}_{\text{k}}+{\text{b}}_{\text{1}}(\text{Teeth})+{\text{b}}_{2}(\text{MS})+{\text{b}}_{3}(\text{COVF})+{\text{e}}_{\text{ijkl}}$$

where μ is the overall mean, FYS_i_, BG_j_, and Sex_k_ are fixed effects; b_1_, b_2_, and b_3_ are linear regression coefficients; and e_ijkl_ is the residual, assumed to be normally distributed with mean zero and variance s_e_^2^.

Farm-level clustering was accounted for through the FYS effect, which captures management and temporal variation. Sensitivity analyses including farm as a random effect produced comparable results; therefore, the GLM specification was retained for parsimony and interpretability. The dataset was unbalanced across BGs (e.g., BFMx, n = 13); thus, estimates for small groups should be interpreted with caution.

#### Model diagnostics and estimation

Model assumptions were verified using residual diagnostics, indicating approximate normality and homogeneity of variance. Multicollinearity was assessed using variance inflation factors (VIF<5). Least squares means (LSM) were compared using t-tests, with significance declared at p<0.05. Regression coefficients were used to quantify the effects of Teeth, MS, and COVF. Pearson correlation coefficients among traits were estimated using the PROC CORR procedure in SAS.

## RESULTS AND DISCUSSION

### Descriptive statistics of carcass traits and pricing of fattened cattle

Descriptive statistics for 5,531 fattened cattle revealed substantial phenotypic variation in carcass and pricing traits within the cooperative system (Table 1). Carcass mass traits showed moderate variability, with mean LW of 613.65±85.43 kg (CV = 13.92%) and similar dispersion for HCW and CCW (CV = 15.27% to 15.64%), indicating that carcass mass is a major source of biological and economic variation, influenced by genetic background, physiological maturity, and management conditions (p<0.001; Tables 2–4) [20].

In contrast, carcass yield traits were relatively stable, with low coefficients of variation for HCP and CCP (5.66% to 5.87%), reflecting consistent dressing performance under standardized practices. Weak correlations between LW and dressing traits (r = 0.04 to 0.09) indicate that increased body mass does not proportionally improve carcass yield, likely due to constraints in tissue partitioning between carcass and non-carcass components [11].

Fat-related traits exhibited the greatest variability, with high coefficients of variation for MS (45.88%) and COVF (71.82%), indicating substantial heterogeneity in adipose tissue deposition. This variability, detailed in Table 1, reflects differences in genotype, physiological maturity, and nutrient partitioning, with fat-related traits showing greater dispersion than carcass mass traits. This pattern underscores the distinct biological regulation of adipose tissue development, particularly the partial independence of intramuscular fat deposition from overall growth, which contributes to carcass differentiation and eating quality [5,21–23].

For pricing traits, PCKG showed low variability (CV = 6.87%), indicating a standardized carcass-based pricing system, whereas PLCAT and PLKG exhibited greater dispersion (CV = 19.68% and 11.52%), primarily driven by variation in carcass mass and fat-related traits.

Overall, these results support a dual-pathway structure of value determination, in which carcass mass drives total economic return (PLCAT), while fat-related traits, particularly marbling, determine price differentiation (PCKG and PLKG).

### Carcass quality

Carcass traits were significantly affected by FYS, BG, sex, and physiological maturity (Teeth) (p<0.001; Table 2), indicating that carcass variation arises from the combined effects of environmental, genetic, and biological factors, as typically observed in tropical crossbred systems [20,24].

FYS accounted for a significant proportion of variation in carcass traits (p<0.001; Table 2), reflecting differences in feeding regimes, forage availability, climatic conditions, and management across farms and seasons [25]. Differences in nutritional management and feed resources across production systems can substantially influence growth performance, carcass composition, and fat deposition in beef cattle [26]. These environmental factors influence growth performance and fat deposition through their effects on energy intake and metabolic efficiency, thereby shaping carcass composition and contributing to variation in economic outcomes [20,21].

BG effects were pronounced, reflecting genetic differences in growth potential and fat deposition. Crossbreds with greater *Bos taurus* influence (e.g., CHAx, WAGx, ANGx) exhibited higher carcass mass and/or marbling, whereas *Bos indicus*-influenced cattle (e.g., BRAx) showed greater dressing efficiency. These patterns are consistent with breed-specific regulation of myogenesis and adipogenesis, in which *Bos taurus* genotypes promote earlier intramuscular fat deposition, while *Bos indicus* genotypes favor prolonged lean growth [27,28]. These differences are further supported by genetic and metabolic mechanisms that control fat partitioning and muscle development [5,9], underscoring the importance of aligning breed composition with market objectives.

Sex effects reflected differences in nutrient partitioning and endocrine regulation. Steers exhibited greater carcass mass due to enhanced muscle accretion, whereas females showed higher subcutaneous fat (COVF), consistent with estrogen-mediated adipogenesis [11,29,30].

Physiological maturity (Teeth) was positively associated with carcass weight, MS, and COVF, reflecting the biological shift from muscle growth to fat deposition with age [23,31]. However, increased maturity may reduce eating quality, particularly tenderness, indicating a trade-off between carcass mass and quality that must be managed through optimal slaughter timing [12].

Overall, carcass quality is determined by the integrated effects of FYS, BG, sex, and maturity, which regulate muscle growth and fat deposition through coordinated biological pathways. These mechanisms underpin the dual mass- and quality-driven value structure observed in carcass pricing.

### Prices of high-quality finished cattle

Pricing within the cooperative system was primarily determined by carcass mass and fat-related quality traits. Model results (Table 3) showed that FYS, BG, physiological maturity (Teeth), MS, and COVF significantly affected all pricing outcomes (PCKG, PLCAT, and PLKG; p<0.0001), indicating that price formation reflects the integrated effects of environmental, genetic, and biological factors.

FYS accounted for a substantial proportion of price variation, reflecting differences in feeding regimes, forage availability, climatic conditions, and market timing across farms and seasons. These factors influence growth performance and adipose tissue deposition through energy intake and metabolic efficiency, thereby linking production conditions to economic outcomes, consistent with tropical production systems [20,22].

BG effects closely paralleled carcass performance, demonstrating a direct biological–economic translation of genotype effects. Crossbreds with greater growth potential (e.g., CHAx) or marbling capacity (e.g., WAGx) achieved higher economic returns, whereas *Bos indicus*-influenced groups showed relatively higher dressing efficiency but lower price premiums. These patterns reflect genotype-specific regulation of muscle accretion and adipogenesis, where *Bos taurus*-derived genotypes favor intramuscular fat deposition and growth, while *Bos indicus* types emphasize lean tissue efficiency [5,9]. However, estimates for small groups (e.g., BFMx; n = 13) should be interpreted cautiously due to limited precision.

Regression analysis further clarified trait–price relationships. Physiological maturity (Teeth) increased total value (PLCAT) via higher carcass weight but reduced price efficiency (PLKG), indicating diminishing returns per unit weight with advancing age [12]. In contrast, MS exerted the strongest positive effect on pricing, confirming its central role in value differentiation and consumer preference [3,5]. COVF also contributed positively, reflecting its role in carcass protection and grading, although excessive fat may reduce efficiency due to trimming losses.

Sex influenced pricing primarily through carcass mass, affecting PLCAT and PLKG but not PCKG, indicating that pricing is driven mainly by carcass attributes rather than demographic classification.

Overall, pricing variation reflects two complementary pathways: carcass mass determining total return (PLCAT) and fat-related traits, particularly MS, determining price premiums (PCKG and PLKG). These findings provide a quantitative basis for optimizing breed composition, management, and slaughter timing within cooperative beef production systems.

### Regression coefficients of carcass quality and prices of quality fattened cattle

Regression analysis quantified the biological and economic pathways linking animal traits to carcass performance and pricing outcomes (Table 4), extending the dual mass-driven and quality-driven framework. Physiological maturity (Teeth) significantly increased carcass mass, with each additional pair associated with higher LW (+15.28 kg) and CCW (+7.93 kg), reflecting continued muscle accretion with advancing age [32]. In contrast, effects on dressing percentage (HCP and CCP) were negligible, indicating that maturity primarily influences absolute carcass mass rather than proportional yield.

Maturity also promoted fat deposition, increasing MS (+0.10 per unit) and COVF (+0.03 cm per unit), consistent with the shift from muscle accretion to adipogenesis during later growth stages [33,34]. This biological transition, supported by mechanisms of nutrient partitioning and lipid development [5], resulted in increased total value (PLCAT: +1,290.71 THB/head) but reduced pricing efficiency, as indicated by declines in PCKG (−0.53 THB/kg) and PLKG (−0.39 THB/kg). This trade-off reflects both market preferences for younger animals with superior eating quality and pricing structures that discount advanced maturity [12,35].

Among quality traits, MS exerted the strongest economic effect. A one-unit increase in MS increased PCKG by 12.34 THB/kg and PLCAT by 5,889 THB/head, confirming that intramuscular fat is the primary driver of price differentiation in quality-based markets [18]. This is consistent with evidence that marbling governs eating quality and consumer willingness to pay through biological pathways largely independent of growth [3,5]. In contrast, COVF had a smaller but significant positive effect (+0.69 THB/kg per cm), reflecting its role in carcass grading and chilling protection, although excessive fat may reduce efficiency due to trimming losses [14,23].

Overall, carcass mass, largely driven by maturity, determines total economic return, whereas fat-related traits, particularly MS, drive price premiums independent of carcass size. The decline in pricing efficiency with increasing maturity highlights the importance of optimizing slaughter timing to balance growth and carcass quality, providing a biologically grounded framework for market-oriented production strategies.

### Effect of breed group

BG was a major determinant of carcass performance, reflecting substantial genetic heterogeneity among crossbred populations (Table 5). Differences were most pronounced for carcass mass traits. CHAx exhibited the highest LW (611.74 kg) and CCW (338.52 kg), whereas BRAx showed lower LW (565.49 kg) and HOLx the lowest CCW (319.33 kg), confirming superior growth potential of CHAx-type crossbreds in systems targeting heavier carcasses [21,22].

In contrast, carcass yield followed an inverse pattern. BRAx achieved the highest dressing percentages (HCP: 58.05%; CCP: 56.20%), whereas HOLx showed the lowest CCP (52.48%). These differences reflect variation in body composition, with *Bos indicus*-influenced cattle exhibiting greater lean tissue efficiency and lower non-carcass components than dairy-influenced genotypes [36].

Fat-related traits showed marked breed-specific variation. WAGx exhibited the highest MS (2.52), followed by ANGx and HOLx (~2.07), whereas BRAx showed the lowest MS (1.23), confirming the strong genetic predisposition of Wagyu-derived cattle for intramuscular fat deposition [7,8]. This is consistent with evidence that genetic regulation of adipogenesis and lipid metabolism underlies breed differences in marbling development [5,9]. In contrast, ANGx exhibited the greatest COVF (1.24 cm), whereas HOLx showed the lowest (0.66 cm), indicating divergent fat partitioning between intramuscular and subcutaneous depots.

Overall, BG defined distinct production profiles: CHAx for carcass mass, BRAx for dressing efficiency, WAGx for marbling, and ANGx for subcutaneous fat deposition. These differences reflect underlying variation in growth biology and nutrient partitioning, which jointly determine carcass composition and economic value. However, estimates for smaller groups (e.g., BFMx; n = 13) should be interpreted with caution. Collectively, these results highlight trade-offs between growth, yield, and fat deposition and provide a biological basis for the mass- and quality-driven pricing mechanisms described subsequently.

### Effect of breed group on the pricing of quality fattened cattle

**Figure 1:**
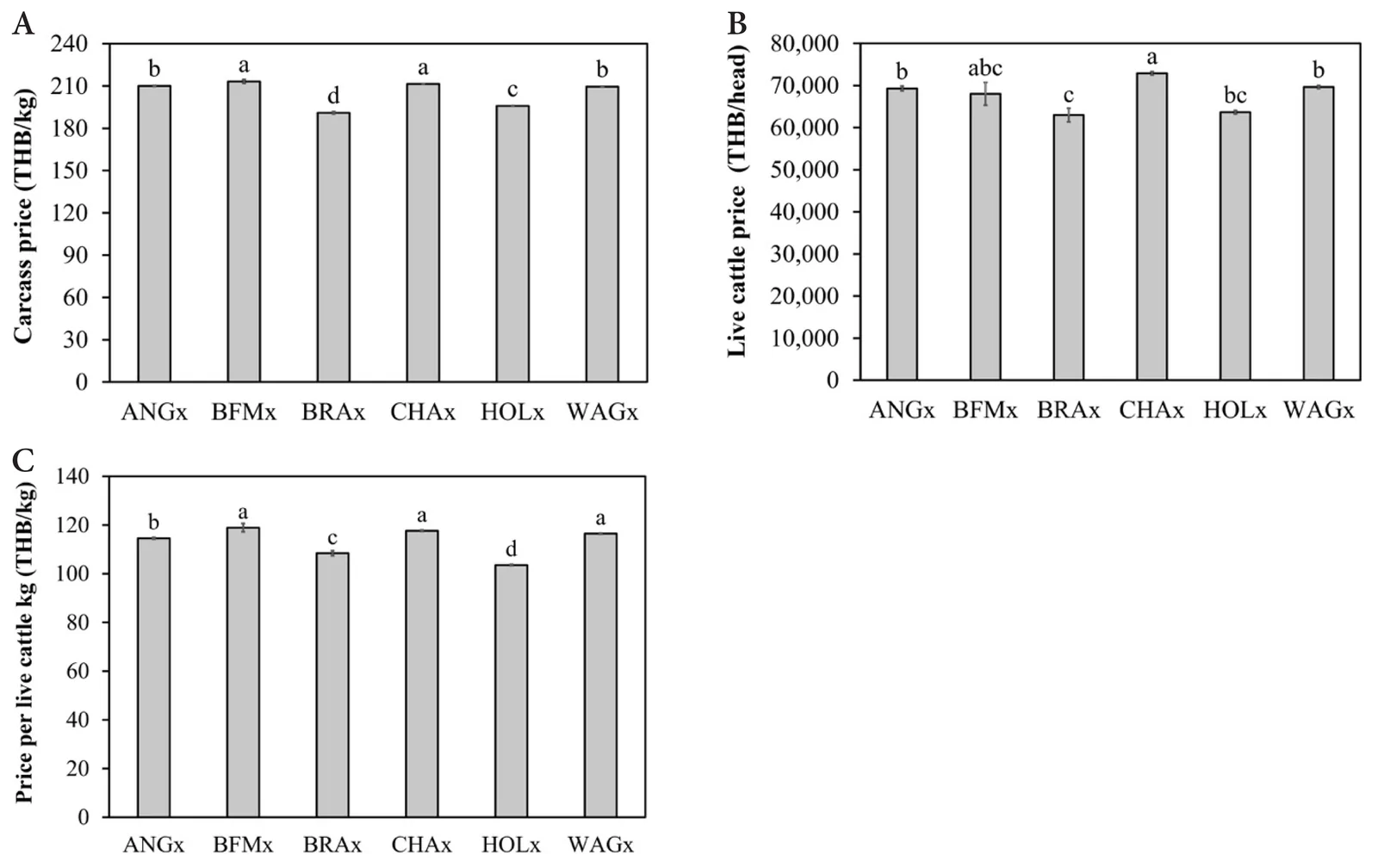
Least squares means and standard errors for (A) carcass price, (B) live cattle price, and (C) price per live cattle kg by breed group. ^a–d^ Least squares means with different superscripts differ (p<0.05). ANGx, Angus crossbred; BFMx, Beefmaster crossbred; BRAx, Brahman crossbred; CHAx, Charolais crossbred; HOLx, Holstein crossbred; WAGx, Wagyu crossbred.

BG significantly influenced pricing traits (Figure 1), reflecting underlying differences in carcass mass and fat-related quality. CHAx exhibited the highest PCKG (211.43 THB/kg) and PLKG (117.64 THB/kg), followed by WAGx, indicating that both growth potential and marbling contribute to price formation within the cooperative grading system [20,22]. Estimates for BFMx should be interpreted cautiously due to the small sample size (n = 13).

Total economic value (PLCAT) was primarily driven by carcass mass, with CHAx achieving the highest value (72,907.67 THB/head), consistent with its superior growth performance [20]. Comparable PLCAT between HOLx and BRAx suggests compensatory effects between carcass weight and fat-related traits, whereby differences in carcass composition may offset each other in determining final value [23].

Breed-specific pricing patterns were consistent with biological differences in tissue deposition. WAGx received higher price premiums due to superior marbling, reflecting enhanced intramuscular adipogenesis [7,8] and its genetic regulation [5,9]. In contrast, CHAx derived economic advantage mainly from greater carcass mass associated with muscle accretion [21,28], whereas BRAx maintained competitive total value through higher dressing efficiency despite lower unit prices [36]. These results support two interacting pathways of value formation: a mass-driven pathway (carcass weight) and a quality-driven pathway (marbling).

Although production cost data were not available in this study, breed-group differences in growth rate, feed efficiency, fat deposition patterns, and finishing duration are likely to influence input requirements and overall production efficiency [14,23]. These factors may lead to variation in cost structure and profitability among BGs. Therefore, optimization of BG should consider both carcass value (mass and quality) and expected production efficiency under specific production conditions. Overall, BG is a key determinant of pricing outcomes, linking genetic background to carcass composition and market value within cooperative production systems.

### Effect of sex

Sex significantly influenced carcass composition and economic outcomes, primarily through differences in growth dynamics and nutrient partitioning (Table 6). Steers exhibited greater carcass mass and yield (LW, HCW, CCW, HCP, CCP) and slightly higher MS, resulting in higher total economic value (PLCAT) and value per unit live weight (PLKG). In contrast, females showed greater COVF, indicating a higher propensity for subcutaneous fat deposition.

These differences reflect sex-specific physiological regulation of tissue development. Androgen-associated anabolic activity in steers promotes muscle accretion, whereas estrogen-mediated processes in females favor adipose tissue deposition, particularly in subcutaneous depots [11,37–39]. This pattern is consistent with differential nutrient partitioning, where energy is preferentially directed toward lean tissue growth in steers and lipid deposition in females, and is further supported by interactions between hormonal regulation and metabolic pathways controlling adipogenesis [5,9].

In the present study, all male animals were castrated (steers), and no intact males were included. Despite observable differences in carcass traits between sexes, sex had minimal influence on price per unit carcass weight (PCKG), indicating that cooperative pricing is primarily determined by carcass quality attributes rather than sex per se. This is consistent with previous studies showing that market valuation depends mainly on carcass characteristics, while sex effects are expressed indirectly through growth performance and fat distribution [14,40]. Accordingly, the economic advantage of steers in this study is largely attributable to greater carcass mass rather than intrinsic price premiums.

Overall, sex-related variation operates through biological mechanisms regulating muscle growth and adipose deposition, complementing breed and maturity effects. These interactions reinforce the dual mass-driven and quality-driven pathways of value determination identified in this study and provide a physiological basis for the relationships among maturity (Teeth), fat-related traits (MS and COVF), and pricing outcomes in cooperative beef production systems.

### Pearson correlation coefficients between carcass quality traits and fattened cattle prices

**Figure 2:**
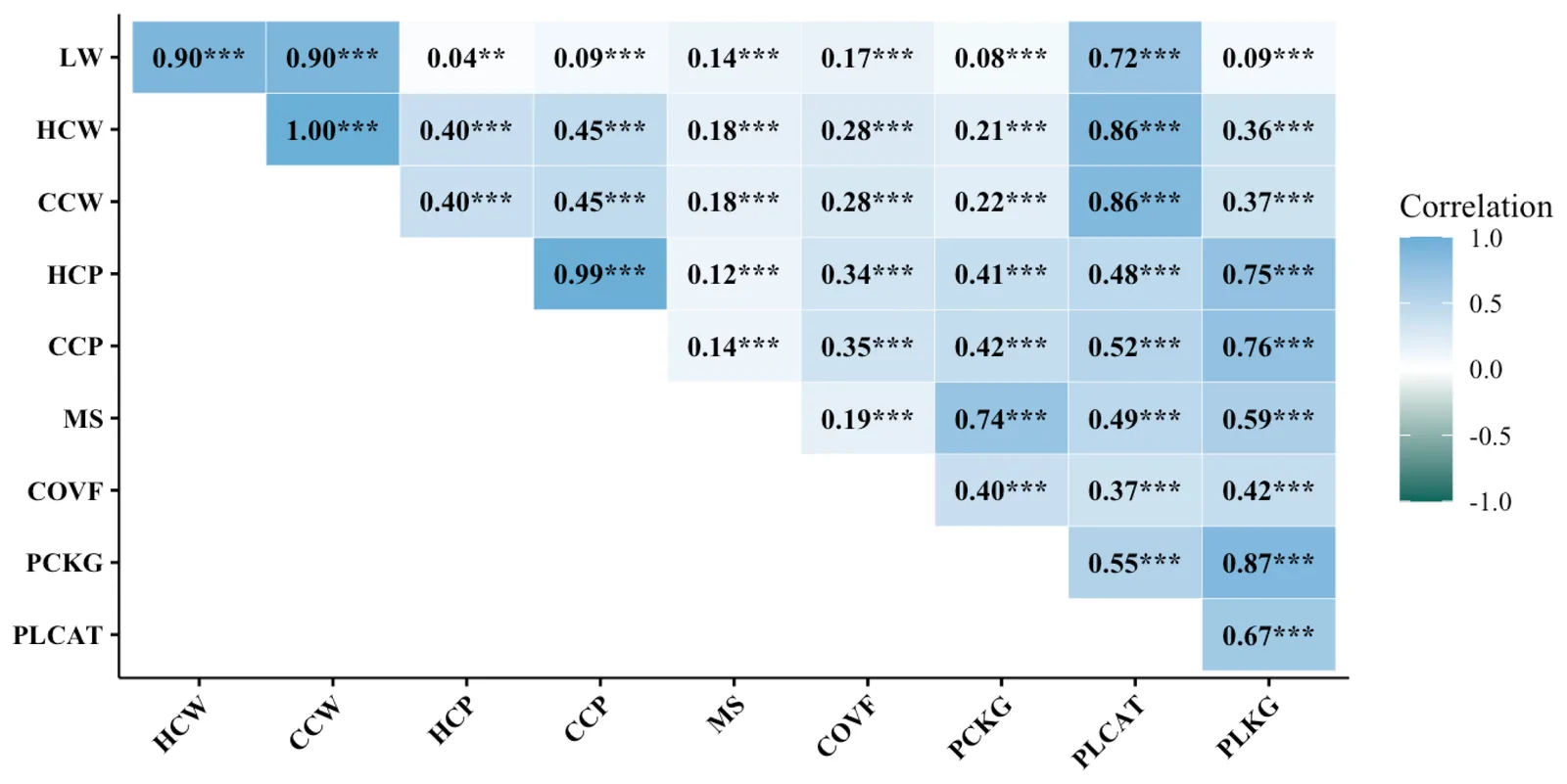
Correlation coefficients between carcass quality and price traits of quality fattened cattle. ** p<0.01, *** p<0.001. LW, live weight; HCW, hot carcass weight; CCW, cold carcass weight; HCP, hot carcass percentage; CCP, cold carcass percentage; MS, marbling score; COVF, cover fat thickness; PCKG, carcass price; PLCAT, live cattle price; PLKG, price per live cattle kg.

Pearson correlation analysis (Figure 2) revealed structured relationships linking carcass traits to pricing outcomes, indicating that economic value is governed by distinct but interconnected biological pathways.

LW showed very strong positive correlations with HCW and CCW (r = 0.90), confirming efficient conversion of body mass into carcass mass. LW was also strongly associated with total economic return (PLCAT; r = 0.72), indicating that carcass mass is a primary driver of overall value. In contrast, correlations between LW and dressing traits (HCP and CCP) were weak (r = 0.04 to 0.09), suggesting that increases in body size do not proportionally improve carcass yield, likely due to constraints in tissue partitioning between carcass and non-carcass components [11].

Dressing traits (HCP and CCP) were almost perfectly correlated (r = 0.99), reflecting their shared biological and mathematical basis and indicating consistent slaughter conditions across the cooperative system.

MS exhibited weak correlations with carcass mass (r = 0.18), indicating that intramuscular fat deposition is largely independent of overall growth and regulated by distinct biological mechanisms involving genotype, nutrient partitioning, and physiological maturity [5,9]. In contrast, MS was strongly correlated with PCKG (r = 0.74; p<0.0001), confirming its central role in determining price premiums in quality-based markets [20,22].

Collectively, these results support two complementary pathways governing economic value: (1) a mass-driven pathway, where carcass weight determines total return (PLCAT), and (2) a quality-driven pathway, where marbling determines price premiums (PCKG) largely independent of carcass size. This dual-pathway structure is consistent with regression and breed-group analyses and reinforces the biological-economic framework linking growth, fat deposition, and pricing outcomes in cooperative beef production systems.

## CONCLUSION

Carcass value in crossbred cattle under a tropical cooperative system is determined by two pathways: carcass mass driving total return and MS driving price premiums. These reflect biological differences in growth and fat deposition influenced by BG, physiological maturity, and sex. CHAx excelled in carcass mass, WAGx in marbling, and BRAx in dressing efficiency, emphasizing the need to align breed selection with market objectives. Increasing maturity improved carcass weight but reduced pricing efficiency, highlighting the importance of optimal slaughter timing. Overall, MS is the key driver of price differentiation, and carcass value reflects the integrated effects of quantity and quality.

## Figures and Tables

**Table 1:** Descriptive statistics for carcass quality traits and fattened cattle prices

Traits	N	Mean	SD	CV
LW (kg)	5,531	613.65	85.43	13.92
HCW (kg)	5,531	347.78	53.10	15.27
CCW (kg)	5,531	337.54	52.78	15.64
HCP (%)	5,523	56.61	3.20	5.66
CCP (%)	5,530	54.92	3.23	5.87
MS (score)	5,398	2.11	0.97	45.88
COVF (cm)	5,518	1.03	0.74	71.82
PCKG (THB/kg)	5,275	206.06	14.16	6.87
PLCAT (THB/head)	5,440	70,351.78	13,844.78	19.68
PLKG (THB/kg)	5,363	113.79	13.11	11.52

SD, standard deviation; CV, coefficient of variation; LW, live weight; HCW, hot carcass weight; CCW, cold carcass weight; HCP, hot carcass percentage; CCP, cold carcass percentage; MS, marbling score; COVF, cover fat thickness; PCKG, carcass price; PLCAT, live cattle price; PLKG, price per live cattle kg.

**Table 2:** Significance level of factors influencing carcass quality, including live weight (LW), hot carcass weight (HCW), cold carcass weight (CCW), hot carcass percentage (HCP), cold carcass percentage (CCP), marbling score (MS), and cover fat (COVF)

Traits	Factors			

	FYS	BG	Sex	Teeth
LW (kg)	<0.0001	0.0002	<0.0001	<0.0001
HCW (kg)	<0.0001	<0.0001	<0.0001	<0.0001
CCW (kg)	<0.0001	<0.0001	<0.0001	<0.0001
HCP (%)	<0.0001	<0.0001	<0.0001	0.0978
CCP (%)	<0.0001	<0.0001	<0.0001	0.3604
MS (score)	<0.0001	<0.0001	<0.0001	<0.0001
COVF (cm)	<0.0001	<0.0001	<0.0001	0.0026

FYS, farm-year-season of slaughter; BG, breed group; Teeth, pair of permanent incisors.

**Table 3:** Significance level of factors influencing fattened cattle prices, including price of the carcass (PCKG), live cattle price (PLCAT), and price per live cattle kg (PLKG)

Traits	Factors					

	FYS	BG	Sex	Teeth	MS	COVF
PCKG (THB/kg)	<0.0001	<0.0001	0.3430	<0.0001	<0.0001	<0.0001
PLCAT (THB)	<0.0001	<0.0001	<0.0001	<0.0001	<0.0001	<0.0001
PLKG (THB/kg)	<0.0001	<0.0001	<0.0001	0.0001	<0.0001	<0.0001

FYS, farm-year-season of slaughter; BG, breed group; Teeth, pair of permanent incisors; MS, marbling score; COVF, cover fat thickness.

**Table 4:** Regression coefficients (b) of key factors influencing carcass traits and fattened cattle prices

Traits	b (Teeth [pair])	b (MS [score])	B (COVF [cm])
LW (kg)	15.28±1.26**	-	-
HCW (kg)	7.95±0.76**	-	-
CCW (kg)	7.93±0.75**	-	-
HCP (%)	−0.06±0.04	-	-
CCP (%)	−0.03±0.04	-	-
MS (score)	0.10±0.01**	-	-
COVF (cm)	0.03±0.01**	-	-
PCKG (THB/kg)	−0.53±0.08**	12.34±0.09**	0.69±0.11**
PLCAT (THB/head)	1,290.71±162.11**	5,889.01±162.88**	2,154.76±227.23**
PLKG (THB/kg)	−0.39±0.10**	7.69±0.10**	1.01±0.14**

** p<0.01.

LW, live weight; HCW, hot carcass weight; CCW, cold carcass weight; HCP, hot carcass percentage; CCP, cold carcass percentage; MS, marbling score; COVF, cover fat thickness; PCKG, carcass price; PLCAT, live cattle price; PLKG, price per live cattle kg.

**Table 5:** Least squares means and standard errors for carcass quality traits including live weight (LW), hot carcass weight (HCW), cold carcass weight (CCW), hot carcass percentage (HCP), cold carcass percentage (CCP), marbling score (MS) and cover fat (COVF) by breed groups

Traits	ANGx (n = 484)	BFMx (n = 13)	BRAx (n = 46)	CHAx (n = 1,586)	HOLx (n = 1,801)	WAGx (n = 1,601)
LW (kg)	604.11±4.34^ab^	574.24±21.48abc	565.49±12.56^c^	611.74±3.08^a^	608.03±3.17^ab^	600.50±2.69^b^
HCW (kg)	340.55±2.62^b^	324.17±12.98abc	327.99±7.59^bc^	348.72±1.86^a^	329.70±1.92^c^	342.42±1.62^b^
CCW (kg)	330.51±2.60^b^	314.09±12.88abc	317.73±7.53^bc^	338.52±1.85^a^	319.33±1.90^c^	332.53±1.61^b^
HCP (%)	56.20±0.13^c^	57.34±0.63abc	58.05±0.37^a^	56.97±0.09^b^	54.19±0.09^d^	56.94±0.08^b^
CCP (%)	54.52±0.13^c^	55.54±0.63abc	56.20±0.37^a^	55.28±0.09^b^	52.48±0.09^d^	55.27±0.08^b^
MS (score)	2.07±0.05^b^	1.80±0.25^bc^	1.23±0.14^d^	1.70±0.04^c^	2.07±0.04^b^	2.52±0.03^a^
COVF (cm)	1.24±0.04^a^	1.01±0.18abc	0.81±0.10^cd^	0.89±0.03^c^	0.66±0.03^d^	1.09±0.02^b^

a–d Least squares mean with different superscripts differ (p<0.05).

ANGx, Angus crossbred; BFMx, Beefmaster crossbred; BRAx, Brahman crossbred; CHAx, Charolais crossbred; HOLx, Holstein crossbred; WAGx, Wagyu crossbred.

**Table 6:** Least squares means and standard errors for carcass quality traits and fattened cattle prices by sex category

Traits	Castrated males (n = 3,109)	Females (n = 2,422)
LW (kg)	616.93±4.61^a^	571.12±4.94^b^
HCW (kg)	352.41±2.78^a^	318.77±2.99^b^
CCW (kg)	342.07±2.76^a^	308.84±2.96^b^
HCP (%)	57.14±0.14^a^	56.10±0.15^b^
CCP (%)	55.42±0.14^a^	54.34±0.15^b^
MS (score)	2.00±0.05^a^	1.79±0.06^b^
COVF (cm)	0.89±0.04^b^	1.01±0.04^a^
PCKG (THB/kg)	205.09±0.29	205.30±0.31
PLCAT (THB)	71,016.92±586.12^a^	64,490.14±633.28^b^
PLKG (THB/kg)	114.29±0.37^a^	112.22±0.40^b^

a,b Least squares means within a row with different superscripts differ (p<0.05).

LW, live weight; HCW, hot carcass weight; CCW, cold carcass weight; HCP, hot carcass percentage; CCP, cold carcass percentage; MS, marbling score; COVF, cover fat thickness; PCKG, carcass price; PLCAT, live cattle price; PLKG, price per live cattle kg.
